# On-Chip Fluorescence Switching System for Constructing a Rewritable Random Access Data Storage Device

**DOI:** 10.1038/s41598-017-16535-7

**Published:** 2018-01-10

**Authors:** Hoang Hiep Nguyen, Jeho Park, Seungwoo Hwang, Oh Seok Kwon, Chang-Soo Lee, Yong-Beom Shin, Tai Hwan Ha, Moonil Kim

**Affiliations:** 1 Hazards Monitoring Bionano Research Center, Korea Research Institute of Bioscience and Biotechnology (KRIBB), 125 Gwahangno, Yuseong-Gu, Daejeon, 34141 Korea; 2 Korean Bioinformation Center, Korea Research Institute of Bioscience and Biotechnology (KRIBB), 125 Gwahangno, Yuseong-Gu, Daejeon, 34141 Korea; 3 Department of Nanobiotechnology, Korea University of Science and Technology (UST), 217 Gajeongno, Yuseong-Gu, Daejeon, 34113 Korea; 4 Department of Pathobiology, College of Veterinary Medicine Nursing & Allied Health (CVMNAH), Tuskegee University, Tuskegee, AL 36088 USA

## Abstract

We report the development of on-chip fluorescence switching system based on DNA strand displacement and DNA hybridization for the construction of a rewritable and randomly accessible data storage device. In this study, the feasibility and potential effectiveness of our proposed system was evaluated with a series of wet experiments involving 40 bits (5 bytes) of data encoding a 5-charactered text (KRIBB). Also, a flexible data rewriting function was achieved by converting fluorescence signals between “ON” and “OFF” through DNA strand displacement and hybridization events. In addition, the proposed system was successfully validated on a microfluidic chip which could further facilitate the encoding and decoding process of data. To the best of our knowledge, this is the first report on the use of DNA hybridization and DNA strand displacement in the field of data storage devices. Taken together, our results demonstrated that DNA-based fluorescence switching could be applicable to construct a rewritable and randomly accessible data storage device through controllable DNA manipulations.

## Introduction

The concept of recording, storing and retrieving information from DNA molecules was proposed very early on by Mikhail Neiman in the 1960s.^1^ Ever since, DNA has been used as a coding language for several applications such as forensics^2^ and product tagging^3^ prior to use in computing.^4,5^ Throughout the years, abundant techniques for DNA manipulation, storage and multiplication have been established as valuable supporting tools for DNA computing.^6–8^ Throughout the years, the search for alternatives to digital storage devices has recently drawn attention to DNA as an attractive target because DNA owns (three) inherent gold standards for information storage devices including longevity, easily achieved storage conditions and a high-density capacity. Recent works in the field of DNA based storage have gained much attention from scholars around the world.^9–13^ However, most of the current DNA-based storage techniques still do not offer the convenience of random data access and high error-handling efficiency.

In these works, different approaches for information encoding were employed using DNA nucleotides such as (A, C = 0 and T, G = 1) in^10^ or base-3 (trits – digits with the values 0, 1 or 2) in.^11^ Following these approaches, information in binary bit stream format (011001011 etc.) could be represented through a nucleotides strand by way of DNA chemical synthesis. To read that encoded information, DNA sequencing is required in the decoding steps to obtain the nucleotide sequences that encoded for binary bit streams of information. When it comes to large information encoding, a commonly used strategy is splitting large strands of DNA into smaller fragments to overcome the difficulty of long strand synthesis and sequencing. However, this solution creates extra work in the assembly of the data and may further induce unwanted errors. In addition, by splitting large strands of DNA into short fragments, data could not be arranged geometrically and maintain its integrity. In general, nucleotide-based approaches for writing and reading information from DNA which employ DNA synthesis and sequencing are error-prone and are relatively different from reading and writing information into/from magnetic disks, optical disks or memory storage devices. Another limitation of the methods is that in the data decoding steps, bulky and costly sequencing machinery is required. After sequencing, the decoding steps also require the help of customized softwares that are compatible with their encoding algorithm.

In our study, we propose a different approach in which not DNA bases but DNA strands are used as basic units for data storage. By taking advantages of DNA manipulation techniques and diverse assets of microfluidic chip such as portability, easier automation and parallelization, we are able to construct a cheap and energy efficient DNA-based storage device which operates in the same manner as that of computer hard disk drives. Our approach addresses a rewritable and randomly accessible DNA-based data storage system that provides a simple way to encode information by fluorescence on-off switching. Also, flexible data encoding and rewriting scheme was evaluated using simple but powerful processes such as DNA hybridization and DNA strand displacement with an intercalating dye (Sybr Green I). The DNA strand displacement (DSD) in the study refers to the use of competitive hybridization, in which two complementary single strands of nucleic acids are joined to form a double helix. The process initiates at a short single-stranded domain called “toeholds” and progress through a branch migration, finally displaces one or more pre-hybridized strands.^14–16^ The rate of DSD reaction could be quantitatively controlled by changing the length and/or sequence composition of the toeholds,^17–19^ thus the process is a powerful tool for DNA hybridization programming. Developed since the 1970’s,^20^ DSD recently draws great research focus for its applications in DNA computing such as in nucleic acid logic circuits,^21,22^ digital circuits computation,^23^ molecular switches,^24^ cascade amplifiers,^25^ catalytic cycles,^26^ genetic programming and evolvable molecular machines,^27^ kinetically controlled self-assembly of DNA oligomers^28^ and DNA nanotechnology.^29^

One advantage of our fluorescence-switching system is that the data decoding step is simplified by putting the encoded data block under a fluorescence scanner to obtain fluorescence data images. These images are then collected and analyzed for the binary code to retrieve the original input information. The results of this work show that information can be easily encoded, decoded and rewritten with the help of simple DNA manipulation techniques and fluorescence indication. In addition, the issue of geometrical storage of data blocks could be solved using the proposed system.

## Results and Discussion

**Figure 1:**
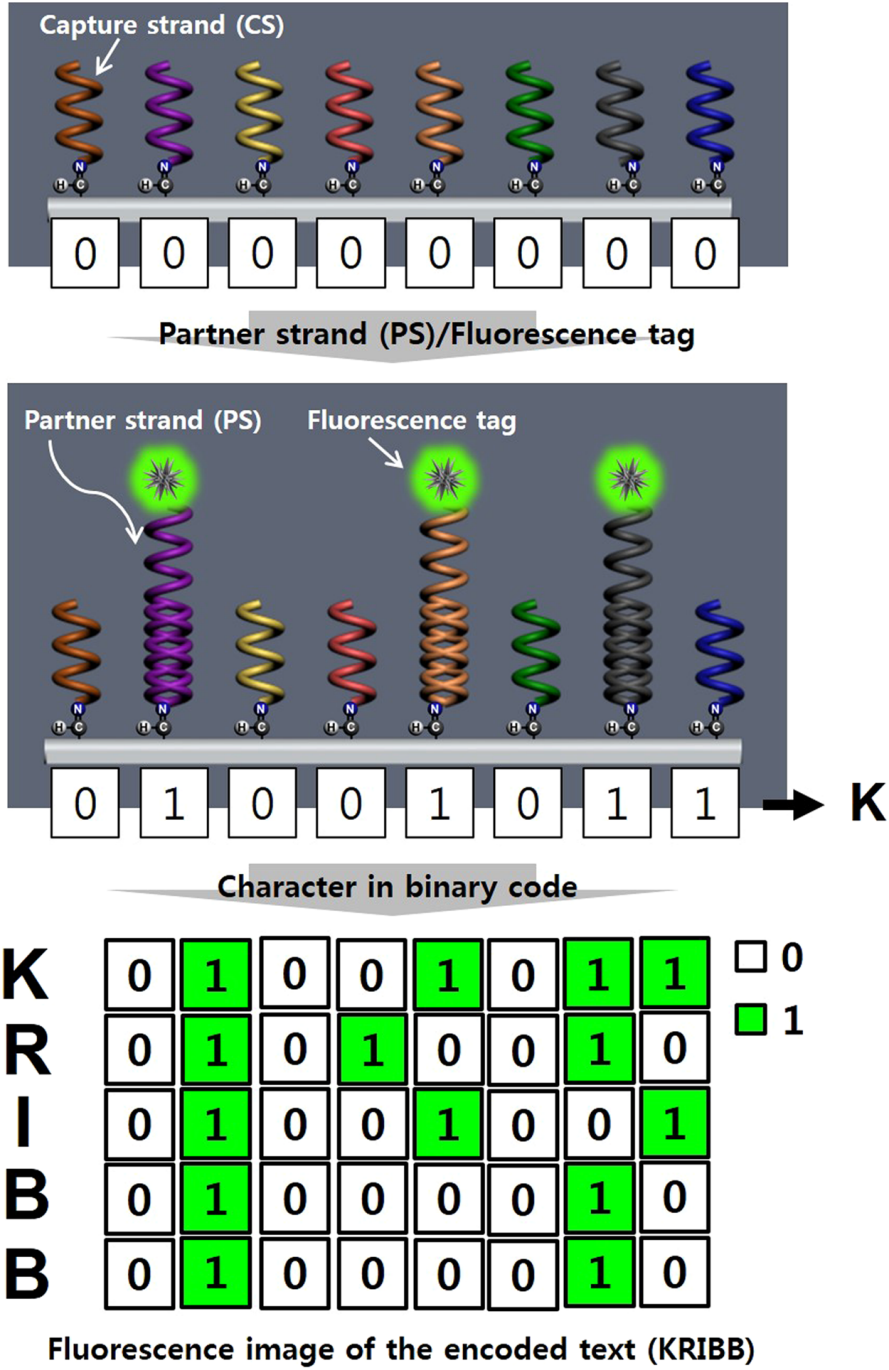
Schematic showing the encoding principle of the text “KRIBB”. The text data is encoded and decoded using the fluorescence signal on-off event with the signal “ON” as the binary bit “1” and the signal “OFF” as the binary bit “0”. The desired text (KRIBB) is converted to binary code following the ASCII to binary conversion rules.

The operating principle of the proposed encoding system is as follows: Digital data (text, image, etc) is converted to binary format at first. We then use fluorescence signal “ON” which results from a duplex DNA and a fluorescent indicator to represents the binary bit “1”, and a fluorescence signal “OFF” corresponding to a single stranded DNA represents the binary bit “0”. The duplex DNA is formed by the hybridization between a capture strand (CS) and a partner strand (PS) meanwhile the single stranded DNA is the capture strand (CS) alone. The current system which uses fluorescence indication as switch is similar to the use of 2 bits, 0 and 1, in the binary base 2, 8-bit number system invented by Gottfried Leibniz in which numeric values are represented by different combinations of 0 and 1 **(**Fig. 1 **)**.

In the current work, we first validate the proposed scheme on a pre-activated glass chip surface and onto a microarray-based microfluidics chip afterwards. The DNA data storage strategy was evaluated by converting a word containing 5 characters (KRIBB) into binary code following the ASCII to Binary conversion, then our encoding procedures was applied for that binary data on to a glass chip surface. Each of the 40 CSs, which is 36-base long amine-terminated single stranded DNA, was immobilized onto the aldehyde-modified glass chip through amine terminus to represent 40 background “0” bits. After that, complementary PSs were immobilized on the corresponding CS spots for “1” bits. A library of nucleotide fragments with a GC content of 50% was randomly generated using random DNA sequence generator (http://users-birc.au.dk/biopv/php/fabox).

**Figure 2:**
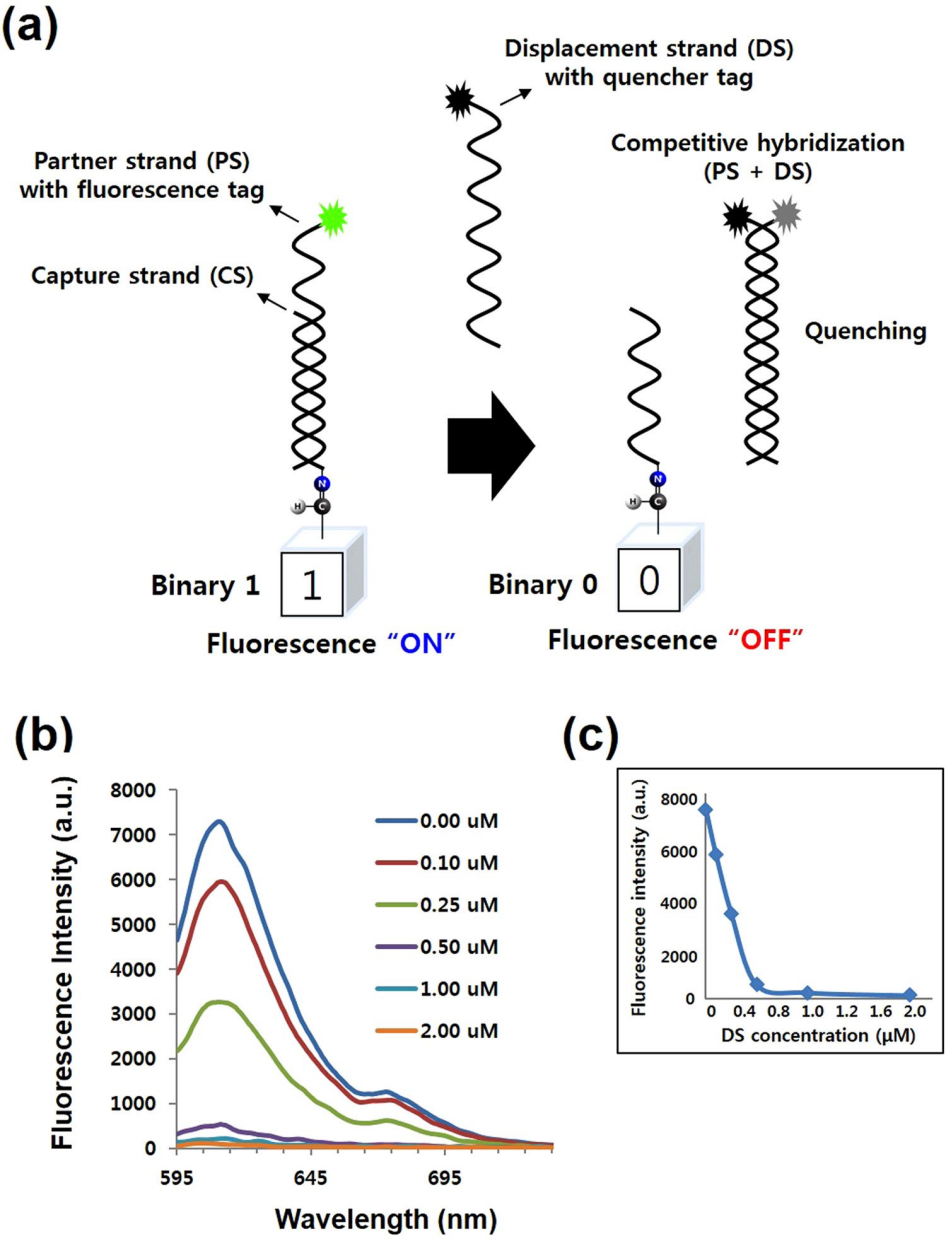
Signal on-off switching using fluorescence and quenching effects. (**a**) Strategy for data rewriting using fluorescence signals with fluorophore (ROX) and quencher (BHQ2) tags. The fluorescence signal “ON” represents the “1” bit, while the signal “OFF” represents the “0” bit. Data rewriting is performed by converting fluorescence signals between “ON” and “OFF” using DNA hybridization and competitive hybridization. (**b**) Changes in fluorescence intensity with various concentrations of DS labeled with quencher. Initial concentration of CS and PS was both 0.5 µM. At 0.25 µM of DS, the fluorescence intensity reduced half of its values. At 1.0 µM and 2.0 µM, the intensity was very weak. (**c**) A calibration curve corresponding to changes in fluorescence intensity.

**Figure 3:**
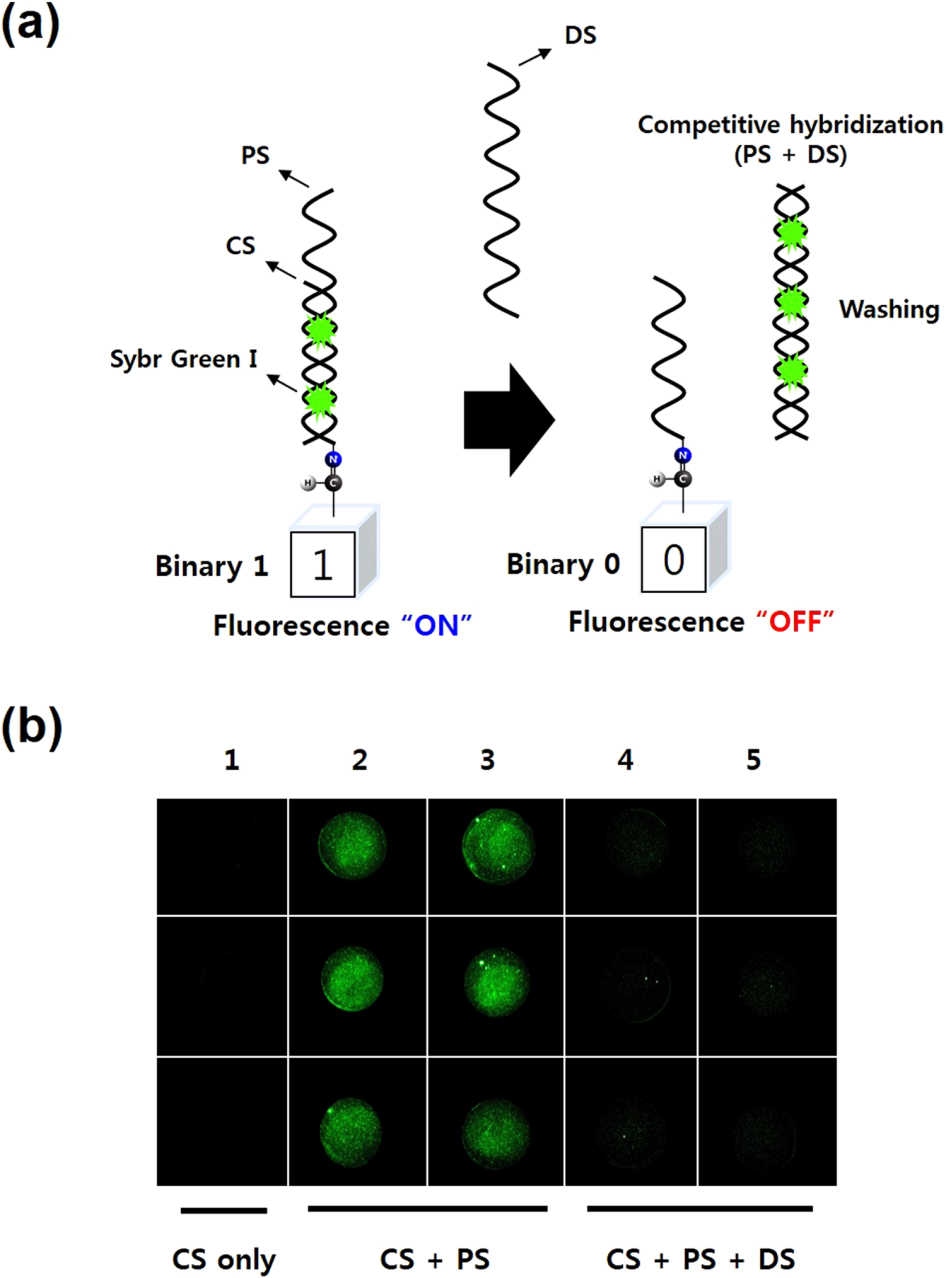
Data rewriting using fluorescence signals with Sybr Green I as an indicator. (**a**) Schematic showing data rewriting at any specific bit by the use of DNA hybridization and DNA strand displacement. (**b**) Fluorescence image showing data rewriting performed on a glass chip. Lane 1: CS only; Lanes 2 and 3: Hybridization of CS and PS; Lanes 4 and 5: Displacement of PS from CS-PS hybridization by addition of DS (200 nM, 500 nM, 1 µM).

Data rewriting studied in this work refers to the conversion of a desire number of binary bit value from 0 to 1 or vice versa. The strategy for data rewriting was carried out by performing DNA hybridization for the “0 to 1” conversion and DNA strand displacement for the “1 to 0” reconversion (Figs 2a and 3a **)**. For DNA strand displacement reactions used in this study, PS was designed to be released from CS-PS complex pre-immobilized onto the surface upon the addition of DS. Successful displacement occurs when DS has a greater affinity for PS thus displacing PS from CS-PS duplex DNA. To accelerate the displacement process, the number of base-pair match between PS and DS must be higher than that between CS and PS, thereby facilitating the formation of a PS-DS duplex DNA. For this reason, the PS used in this work has a 6-base long toehold domain on its 5′ end which help facilitate the process of PS displacement by forming PS-DS hybridization **(**Supplementary Table 2 **)**.

In the initial *in vitro* evaluation test, ROX fluorophore in (ROX)-PS-CS duplex was used as fluorescence-on indicator and BHQ-ROX quenching complex in (BHQ)-DS-PS-(ROX) was used as fluorescence-off indicator (Fig. 2a). Increasing concentrations of DS (0.1, 0.25, 0.5, 1.0 and 2.0 µM) were added to tubes containing 0.5 µM of CS-PS duplex to initiate strand displacement processes. As shown in Fig. 2b and c, a linear correlation were shown between the decreases in fluorescence intensity and the increases in the DS concentration. When the DS concentration reached half that (0.25 µM) of CS-PS, the fluorescence intensity dropped to half compared to the control tube (0.0 µM DS). As DS concentration reaches 0.5 µM, 1.0 µM and 2.0 µM, fluorescence signal decreases approximately 15-fold, 37-fold and 75-fold, respectively compared with that of the control tube. Nevertheless, the use of fluorescence signals with fluorophore and quencher tags is costly. Taking this cost-effectiveness into consideration, Sybr Green I that is known to have strong binding affinity and specificity for double-stranded DNA compared to single stranded DNA can be a good alternative (Fig. 3a). Except for the fact that Sybr Green I is a cheap and commonly used dye for nucleic acid detection, it has also advantage of being able to operate on DNA-modified chip support.

As shown in Figs 2a and 3a, in DNA hybridization, CS and PS are hybridized to form a duplex DNA that turns on fluorescence due to the presence of fluorescent substrate (ROX or Sybr Green I). Meanwhile, in DNA strand displacement, DS displaces PS from pre-hybridized CS-PS duplex to forms PS-DS duplex thus leaving a long single stranded CS on the surface leading to a fluorescence turn off. The CS-PS modified glass slide is prepared following procedures for DNA immobilization and hybridization on glass chip described in the materials and method section. After that, DS solutions of different concentrations (200 nm, 500 nm, 1 uM) were introduced into hybridization spots for displacement processes and left incubated at 37 °C for 1hr. After washing and nitrogen air drying, the chip was scanned for fluorescence image. As shown in Fig. 3b, when amine-modified CS was applied to the aldehyde-functionalized glass slide alone, no fluorescence signal was observed due to extremely weak affinity of Sybr Green I to CS as a single stranded oligonucleotide (Lane 1). Whereas, on CS-PS duplex spots, green fluorescence were seen owing to the intercalation of Sybr Green I into the double stranded DNA (Lanes 2 and 3). There was no or negligible (if any) fluorescence observed on lanes 4 and 5, wherein DS solutions of 3 different concentration displace PSs and remain only CS on the spots. These results demonstrated that data rewriting could be performed through the fluorescence on-off switching system by the use of DNA strand displacement and DNA hybridization.

**Figure 4:**
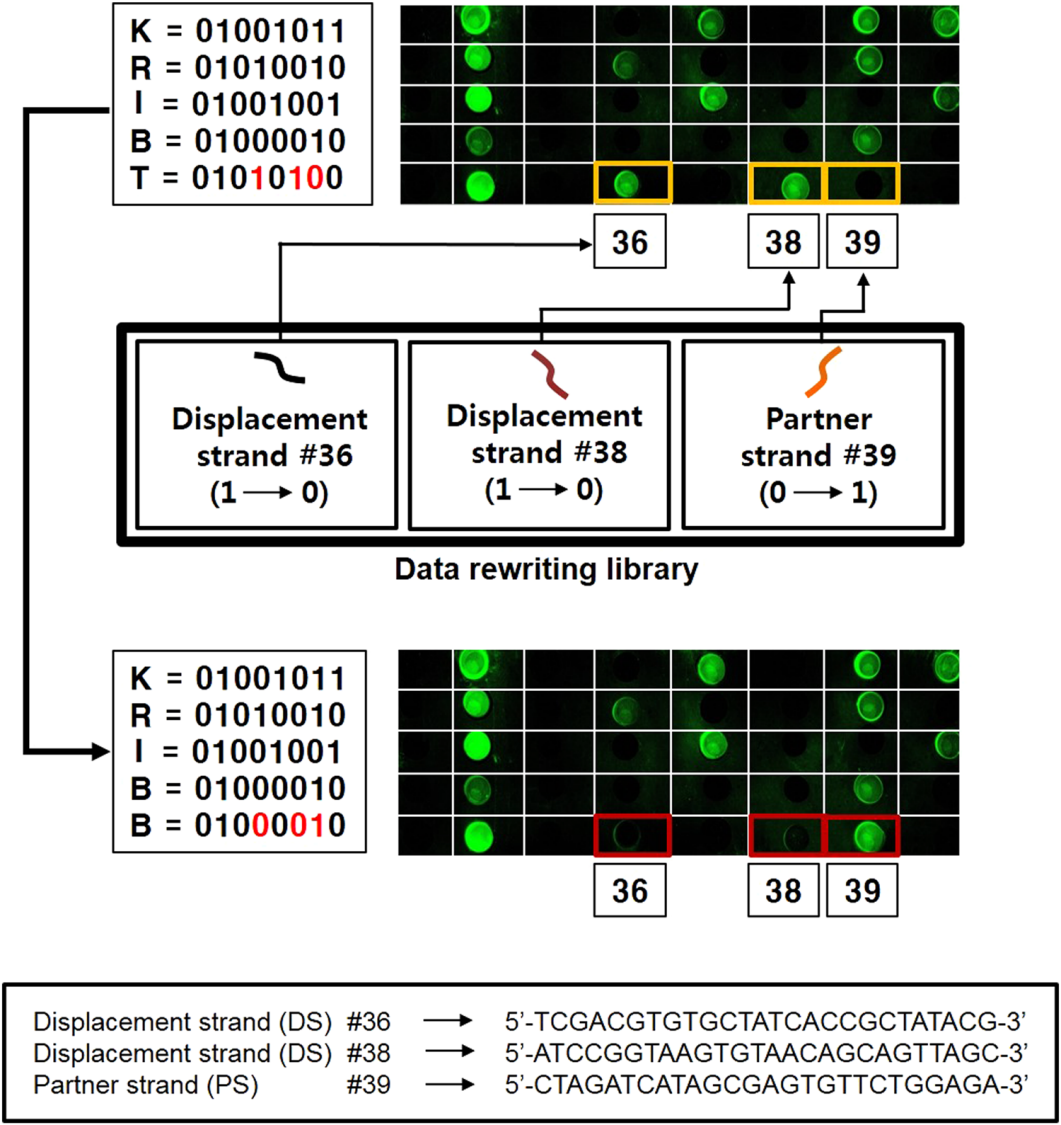
Validation of data encoding and data rewriting on DNA glass chips. Fluorescence image (up) displays the encoding for the erroneous text “KRIBT”. In the image, each fluorescence dot indicates “1” bit, meanwhile dots with no fluorescence representing “0” bits. To repair the typographic in the text (T → B), two of “1” bits at 36^th^ position and 38^th^ position must be converted to “0”, and one “0” bit at position 39 must be converted to the “1” bit. These were achieved by the addition of corresponding DSs on #36 and #38 spots, and addition of corresponding PS #39 on #39 spot for DNA hybridization.

For data rewriting test on a DNA glass chip, we intentionally encode the word “KRIBT” which contains a typographic character (T instead of B) and then apply our rewriting strategy for correction (KRIBT → KRIBB). The encoding of the word KRIBT on the glass chip was made following the encoding principle and procedures in materials and method section. According to the ASCII to binary conversion rules, letter T is represented as 01010100 while letter B is equivalent to 01000010 in binary characters. To repair the error in the text (T → B), two of “1” bit at spot position “36” and “38” on the glass chip must be converted to “0” and one “0” bit at position 39 must be converted to the “1” bit (Fig. 4). The “1” to “0” bit conversions were done using DNA strand displacement by injecting 100 nM of DS #36 and DS #38, which are perfectly complementary to their cognate PSs, and consequently displace the 2 PSs from their duplexes. The “0” to “1” bit conversion was performed by addition of 100 nM of PS #39, leading to double helix formation at position 39. After fluorescence scanning, the obtained glass DNA chip image showed that fluorescence turned “ON” at 36^th^ and 38^th^ spots, and fluorescence turned “OFF” at 39^th^ spot, these results indicated a successful data rewriting process.

**Figure 5:**
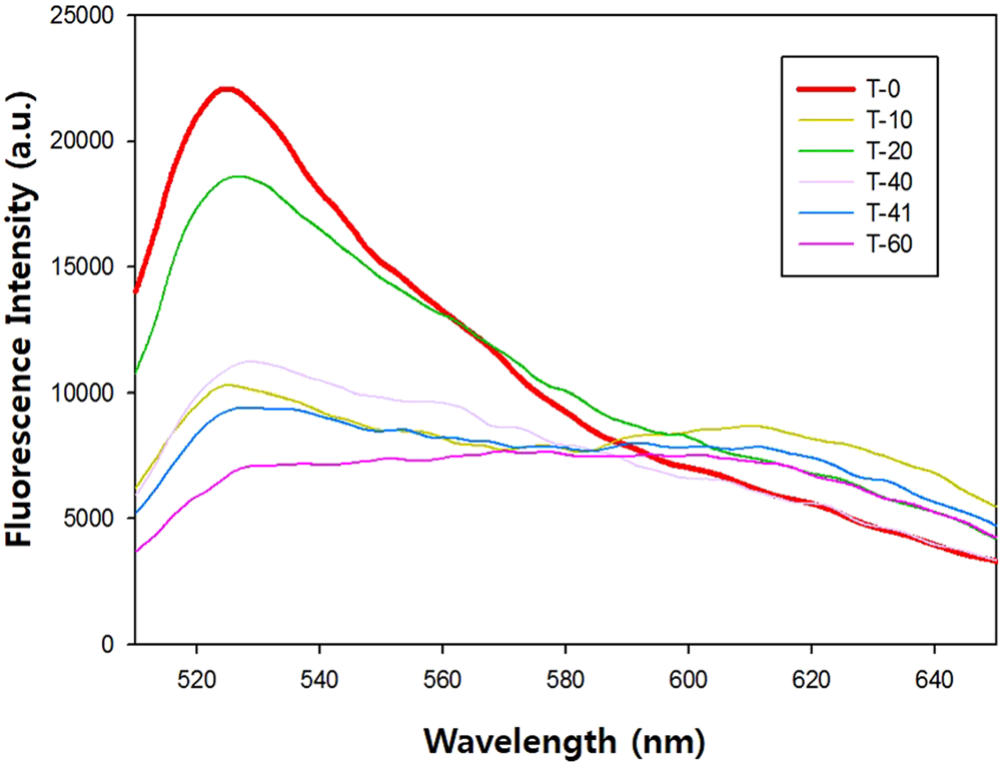
Hybridization efficiency test using Sybr Green, the fluorescence intensity was measured at 525 nm. As the number of mismatches increases, the hybridization efficiency decreases (T-0 > T-20 > T-10 > T-40 ~ T-41 > T-60). T-10 has a single mismatch at the center of the strain which significantly weakened the hybridization, meanwhile T-20 has 2 mismatches distributed distantly from the strand center, and therefore the DS hybridization efficiency is higher than that of T-10.

To test the specificity of the hybridization efficiency used in our scheme with the use of Sybr Green I, we employ a number of mismatched sequences for hybridization efficiency test as shown in Table 1. As a result, Fig. 5 shows that the hybridization efficiency reduces correspondingly (T-0 > T-20 > T-10 > T-40 ~ T-41 > T-60), as the number of mismatches increased. It is noted that T-10 which contains a single mismatch at its center showed very weak hybridization strength, meanwhile T-20 which has 2 mismatches distributed distantly from the strand center, has higher hybridization efficiency than that of T-10.

**Table 1:** Sequences of mismatched oligonucleotides for hybridization efficiency test. The underlines indicate mismatches.

Name	No. of mismatches	Sequence (5′-3′)
Probe	—	CCACATACATCATATTCCCTCATTCAATACCCTACG
T-0	0	TGGAGACGTAGGGTATTGAATGAGGG
T-10	1	TGGAGACGTAGGGTATCGAATGAGGG
T-20	2	TGGAGACGTAGGATATTGAAGGAGGG
T-40	4	TGGAGACGTTGGGTATCTAATAAGGG
T-41		TGGAGACGTAGGACATTGACAGAGGG
T-60	6	TGGAGACGTGTGGCATTCAATTAAGG

**Figure 6:**
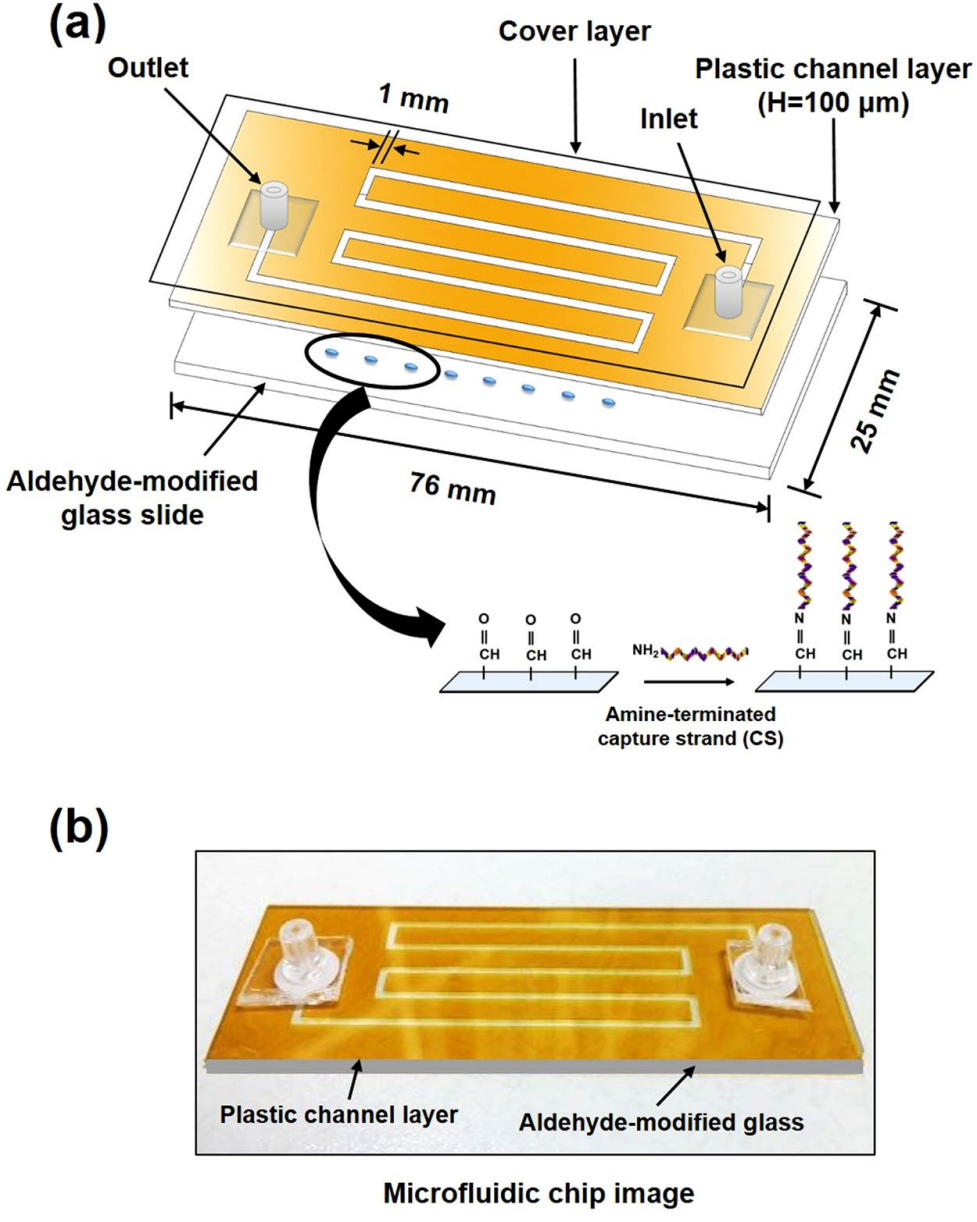
Illustration of the microfluidic chip used for data encoding and rewriting. (**a**) Schematic drawing of the assembled microfluidic device with inlet and outlet connectors. The entire chip consists of 3 layers. The top cover layer provides a seal, and the middle layer made of plastic film containing flow microchannel are placed over an aldehyde-modified glass substrate on which CSs were immobilized. (**b**) Photograph of the film-based microfluidic chip fabricated for the study.

To evaluate the reliability of a rewritable, random-access DNA-based storage system in delivering fluid, we fabricated a microarray-microfluidic device that consists of 3 layers (Fig. 6a and b **)**. The top cover seal and the bottom glass chip were separated by the middle layer with fluidic channels. A detailed fabrication of the device is described in Materials and Methods.

**Figure 7:**
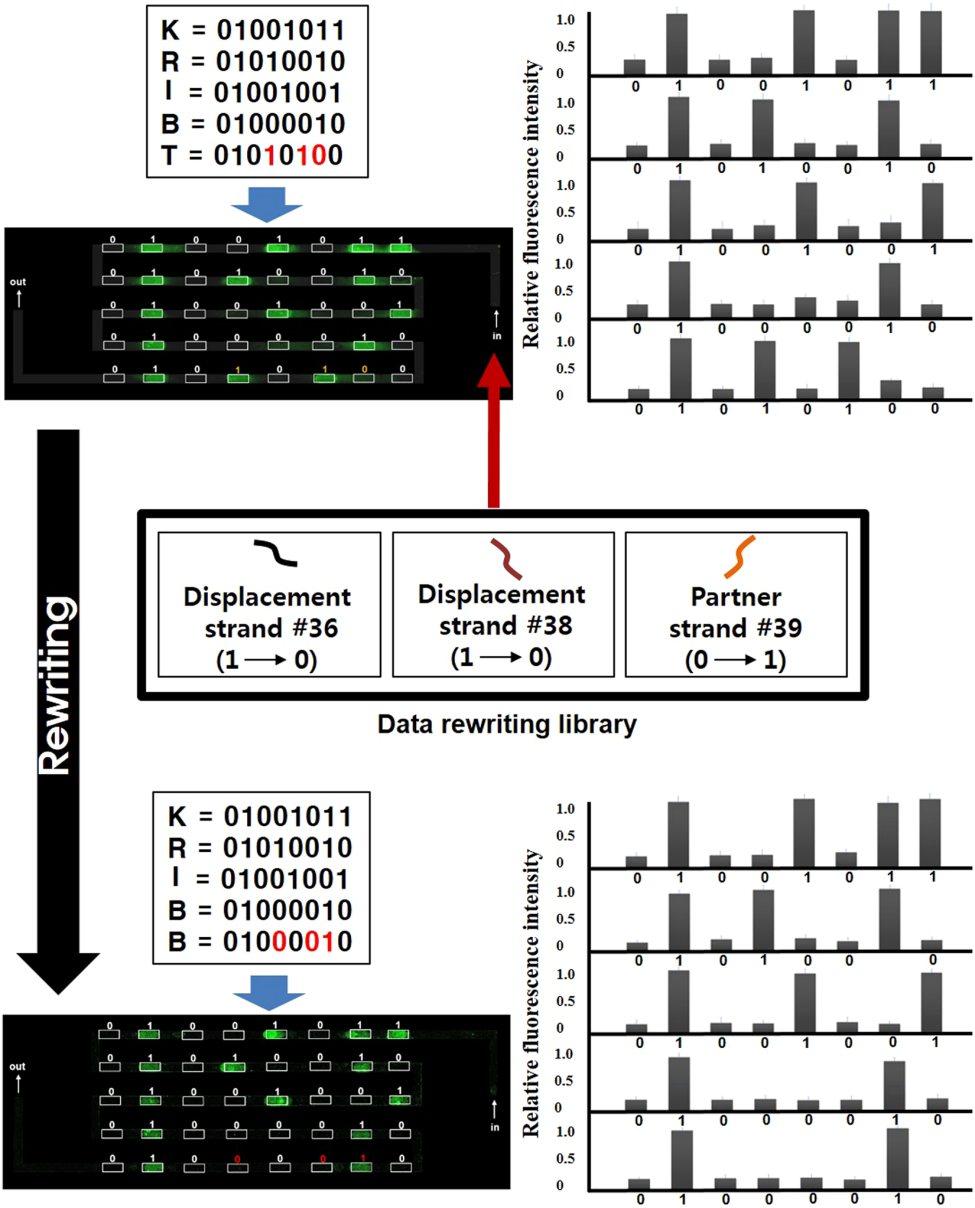
Rewritable DNA data storage system using fluorescence switching on a microfluidic chip. Each letter uses 8 bits per channel to store its information. Fluorescence images of the intentionally made erroneous text KRIBT, and the rewritten text KRIBB are shown after hybridization and strand displacement processes. We set 50% increase in the relative fluorescence intensity on the microfluidic chip as cut-off level for representing the binary 1. Two DSs for #36, #38 spots and one PS for #39 spot were injected through the inlet connector for data rewriting, respectively.

For data rewriting test on the microfluidic chip, the procedure for DNA glass chip was similarly applied with modifications in concentration of CS and PS (See Materials and Methods). After sequential hybridization, for data rewriting, the resulting microfluidic chip was then serially introduced with 2 DSs (10 µM) that displace PSs #36 and #38, and 1 PS (10 µM) that hybridizes with the CS #39. After washing, the channel was injected with 50 × Sybr Green I solution and left incubated for 1 hr prior followed by buffers washing. The microfluidic channel slide was scanned for fluorescent image (Fig. 7). These data confirmed the results obtained from the glass chip test emphasizing the potential of our proposed system on the microfluidic format. The writing, reading, and rewriting of data on this system is rather similar to that of electronic random access memory. Under the current experimental settings, there was no crosstalk between oligonucleotides when applied on the array-based channel as no strange hybridization was observed on fluorescence image.

The decoding of data is simply done by putting the information-encoded glass slide under a fluorescence scanner with conditions set for Sybr Green I (Excitation wavelength of 488 nm and standard blue filter: 508–560 nm). Fluorescence images are then collected and analyzed for binary values which are then converted back to ASCII text lane by lane to retrieve the original input information. Despite a considerable interest in using the current microfluidic system as a rewritable and random-access data storage device. The procedure for the microfluidic hybridization used in this paper, however, is still time and reagents consuming. To shorten the hybridization time for a higher density data storage device, shuttle hybridization method proposed by Cheng-Wey Wei in 2005^30^ could be employed for the next device version. The principle of this method is to use a discrete sample plug to take advantage of the chaotic mixing of droplets. In the paper, the authors successfully obtained data for a 5,000 probe microarray using only 1 µl of the target solution with a hybridization time reduced down to 500 s. To apply this scheme to the current device, we need to design the upper part of the microfluidic chip to have a sealed air chamber at the distal terminal for compressed air storage which helps shuttle the sample solution on injection.

Another challenge in high data density device developments is that the system performance might be limited by non-specific hybridizations between PS-CS or DS-PS which may occur in large oligonucleotide library. Such nonspecific hybridization may be addressed by designing each CS with low sequence similarity and subtracting the intensity of the nonspecifically hybridized signal from the true positive signal. However, by combining DNA strand displacement reaction and DNA hybridization with microfluidic system that has potential to perform thousands of reactions in parallel, our on-chip DNA-based data storage device system based on fluorescence switching could be further developed for higher data storage capability with fast switching and high on-off contrast. For example, the use of a programmable microfluidic platform integrated with high density of oligo arrays (which contains hundreds to thousands array spots) and nano-scale DNA hybridization and DNA strand displacement reactions could enlarge data storage capability of the device. The use of shuttle hybridization as mentioned earlier could improve on-off switching speed, reduce hybridization time and reagents. In addition, error correction codes could be possibly implemented in the next version of the device.

## Conclusions

To the best of our knowledge, this is the first report of the use of DNA hybridization and DNA strand displacement as encoding and rewriting methods in constructing a data storage device. In this study, the feasibility and potential effectiveness of our fluorescence switching-based optical encoding system was evaluated with a series of wet experiments, in which rewriting and randomly accessing of data was successfully achieved on a microfluidic chip. As a combination of biology and computer science, our method offers not only an alternative for information storage device but also an approach to overcome the obstacle of current existing read-only encoding methods. Besides, with the use of fluorescence switching and DNA manipulation techniques, the current approach eliminates the need for expensive and bulky sequencing machinery as well as error-prone long DNA strand synthesis, and allowed for simple and easy error handling of data. Although this study demonstrated a minuscule amount of encoded text data, at least it is demonstrated that a DNA-based fluorescence switching system could be potentially useful to fabricate a rewritable and randomly accessible digital information storage device on microfluidic chip platform. Furthermore, the extent of this work could has considerable value in data storage as a microarray-based framework could possibly have hundreds to thousands of array spots which could enlarge the data density of our proposed device. In this context, studies on reducing the possibility of signal interference which caused by crosstalk between arrays in large oligonucleotide library in the microfluidic channels environment are underway.

## Materials and Methods

### Reagents & materials

Aldehyde-modified glass slides (SuperAldehyde) were obtained from Arrayit Corporation (Sunnyvale, CA), and Glycine was purchased from Sigma-Aldrich (St. Louis, MO). Saline-sodium citrate (20 × SSC solution) was obtained from Ambion Corporation (Austin, TX). Sybr Green I was purchased from Lonza Rockland, Inc. (Rockland, ME). All reagents were of analytical grade if not further specified. Ultrapure water with an electrical resistance larger than 18.2 MΩ was obtained with Milli-Q system from Milipore Corporation (Billerica, MA), and used throughout the study. The domain sequences used in this study were synthesized and HPLC purified by Bioneer Corporation (Daejeon, Korea). The sequences of oligonucleotides used in this study were listed in Supplementary Tables 1 and 2.

### In-tube DNA hybridization

The double-stranded DNA (dsDNA) for in-tube strand displacement test was prepared by mixing equal concentration of CS and PS in the hybridization buffer (10 mM Tris-HCl, 50 mM NaCl, and 1 mM EDTA, pH 7.8). The mixture was heated to 90 °C for 5 mins, cooled slowly 20 °C at a constant rate over a time course of 2 hrs, and finally stored at 4 °C for further use.

### Fluorescence measurement

For in-tube strand displacement test, all the fluorescence measurements were performed using a quartz cuvette (with a path length of 10 mm and an inner width of 1 mm) and a fluorescence spectrometer (FluoroMate FS-2-SCINCO) from Scinco Co. Ltd. (Seoul, Korea). For ROX and BHQ labeled DNA, the emission spectra were collected from 595 to 800 nm, with an excitation wavelength of 588 nm at room temperature. The excitation and emission slit widths were set at 3 nm, with a scanning speed at 600 nm/minute. The fluorescence intensity at 607 nm was chosen as the optimal signal for ROX fluorophore. For tests on a DNA chip and a microfluidic device, the fluorescence images are obtained using GenePix 4200 A Array Scanner from Axon Instrument Inc. (Union City, CA) at a resolution of 10 µM, PMT power is 70%, excitation wavelength is 488 nm. Sybr Green I is known to give emission at 525 nm, therefore the standard blue filter (508–560 nm range) was chosen.

### Fabrication of a microfluidic chip

The film-based microfluidic chip was designed with the assistance of AutoCAD computer software from Autodesk, Inc. (Santa Barbara, CA). The fabricated microfluidic chip consists of a bottom glass slide and 2 film layers which are: A transparent polyethylene terephthalate (PET) film and a double sided adhesive polyimide film (PI) which were all purchased from SKC, Inc. (Covington, GA) and cut using plotting cutter machine (FC4600C-50 pro) from Graphtec Corporation (Irvine, CA). The PI film layer is fabricated to contain fluidic channels which are 1 mm in width and 100 μm in height respectively. The microchannel has one inlet and one outlet made of plastic connectors to let samples going in and out of the channels. The inlet hole diameter is designed to fit a 100 µl pipette tip hence sample injection is made using pipette. The microarray-microfluidic hybrid chip is made by attaching the PET film on top of the upper adhesive side of the PI layer, and the bottom adhesive side of PI layer over the micro-array glass slide. When the 3 parts are completely piled up on top of each other, a microfluidic chip is constructed with total dimensions of 76 mm × 25 mm in size.

### DNA immobilization

Forty amine modified CSs (Supplementary Table 1) were dissolved in 1× PBS solution to a final concentration of 100 nM (for glass slide test) or 30 µM (for microfluidic chip test). Spotting was performed manually by pipetting onto 40 predefined spots on the glass slide to produce a microarray. After spotting, the slide was incubated in a 37 °C incubator for 1 hr followed by washing with serial buffers (Buffer 1: 1× SSC, 0.1% SDS; Buffer 2: 0.1× SSC, 0,05% SDS; Buffer 3: 0.1× SSC; Buffer 4: Deionized water) and nitrogen drying. A blocking solution of 150 mM glycine was applied on the microarray surface by immersing the DNA glass chip into glycine solution or pipetting into the microfluidic chip channel (after microfluidic chip stacking) and left incubated at 37 °C for another 1 hr. Finally, the surface was rinsed with the washing buffers and copious amounts of water and then carefully dried by nitrogen air before hybridization.

### Glass chip hybridization and static microchannel hybridization

On the DNA glass chip, after CS immobilization, 1× PBS buffer were applied on “0” bit spots, whereas for “1” bit spots PSs (100 nM) and 20× Sybr Green I were applied to corresponding CSs for hybridization and left incubated at 37 °C for 1 hr. After serial buffers washing and nitrogen drying, the chip is ready for fluorescence scanning. On the microfluidic chip, after CSs immobilization and chip stacking, each of the 14 PSs to a final concentration 10 μM was sequentially injected into the microchannel using a pipette through the inlet connector and left for incubation at 37 °C for 40 mins. Due to the capillary effect within the microchannel, the injection was rapid and performed with a normal pipetting force. Following hybridization, the slide was washed by pipetting in serial washing buffers: 1× SSC, 0.1% SDS for 10 mins; 0.1× SSC, 0.05% SDS for 5 mins; 0.1× SSC for 5 mins twice. Next, the microfluidic channel was incubated with 50× Sybr Green I solution at room temperature for 60 mins, and then washed with the same serial washing buffers. For data rewriting, the prepared microfluidic chip after sequential hybridization was then serially introduced with 2 displacement strands (DSs #36 and #38) that form a new double helix with PSs #36 and #38, respectively, and 1 partner strand (PS #39) that hybridizes with CS #39. After washing, the channel was injected with 50× Sybr Green I solution, left for incubation in 1 hr prior to washing with buffers and fluorescence scanning.

## Electronic supplementary material

Supplementary Table
