# Supplementary material for: On-Chip Fluorescence Switching System for Constructing a Rewritable Random Access Data Storage Device
Source: Sci Rep. 2018 Jan 10;8:337. doi: 10.1038/s41598-017-16535-7 (PMC5762669; doi:10.1038/s41598-017-16535-7)
Supplement: Supplementary file 1 — Supplementary Table [file 41598_2017_16535_MOESM1_ESM.pdf]

On-Chip Fluorescence Switching System for Constructing a Rewritable Random Access Data Storage Device

Hoang Hiep Nguyen[1,3,§], Jeho Park[1,3,§], Seungwoo Hwang[2], Oh Seok Kwon[1,3], Chang-Soo Lee[1,3], Yong-Beom Shin[1,3], Tai Hwan Ha[1,3,*], and Moonil Kim[1,3,4,*]

[1]Hazards Monitoring Bionano Research Center, [2]Korean Bioinformation Center, Korea Research Institute of Bioscience and Biotechnology (KRIBB), 125 Gwahangno, Yuseong-Gu, Daejeon 34141, Korea; [3]Department of Nanobiotechnology, Korea University of Science and Technology (UST), 217 Gajeongno, Yuseong-Gu, Daejeon 34113, Korea; [4]Department of Pathobiology, College of Veterinary Medicine Nursing & Allied Health (CVMNAH), Tuskegee University, Tuskegee, AL 36088, USA

[§]These authors contributed equally to this work.

Corresponding Authors:

*E-mail: taihwan@kribb.re.kr, kimm@kribb.re.kr

Table 1. List of sequences of 40 capture strands used in this study.

Spot	Sequence
1	5'-Amine-TTGTATATGTATCAGTCTTGACAAGCTGCAGTGTAA-3'
2	5'-Amine-TCTGTATAACTCCAGTGGTTAACACCAGATGGAGAC-3'
3	5'-Amine-TAATTGTCAGCTCAACTCATGATGGCAATGTCGACT-3'
4	5'-Amine-AACAGGCTCACTCTACAACCTGGTTCTTAATTATTG-3'
5	5'-Amine-TTGCTGAACCATAAGTCCTCTAGCGACACTTGTTGG-3'
6	5'-Amine-ACATCGCTACTTCGGAGTCCTGTCATTCGGTTAGTC-3'
7	5'-Amine-TACTTCGAACGTCACCGAATACGCACATGCAGGTCC-3'
8	5'-Amine-CCTGTGTGGAGCTCAGTTGTACACTTAGCAAGGTAG-3'
9	5'-Amine-GCATCTTGGCATTCACCTGTGTTATAAGTCTGTATA-3'
10	5'-Amine-GGATGCGCCTGCCTGAATATCAAGATTCATCCTGCT-3'
11	5'-Amine-GAACGTGGTAGGTAGCATGATTCGTCTGCATCGTGC-3'
12	5'-Amine-GAACCTCAAGCGTTCTGTGCGGTGCTGTGCGGAGAT-3'
13	5'-Amine-TGCTGTCGTCAGACTCCTATAGCCTTCACCTTCCAC-3'
14	5'-Amine-CCTAGGTGATCGGTCGGCGCCGACTCCTATGAACTC-3'
15	5'-Amine-TACCACGGTACGCTCAACGCGCACGTGAGATAGACT-3'
16	5'-Amine-TGGTATAAGCTGAACGGCCTCCGCCATGGCGCGATA-3'
17	5'-Amine-AATGGACCTAATGACTATACCGTAAGAGGATGAACA-3'
18	5'-Amine-CCGCTACAGATGTCGACTATGCGTTAACTCTTCGTC-3'
19	5'-Amine-GAGCGTACGAACGCTATCGACTTACGCTCATGCTCT-3'
20	5'-Amine-AGATCTATTGATTACGTGAATTGAACAACTTCGGTC-3'
21	5'-Amine-GTAGCATCGCCAGTTAGACTGTGAGAGGTTCCGAAG-3'
22	5'-Amine-GGTTGCGTCGTGATTACGCGACGTACTTATCTTACA-3'
23	5'-Amine-GTAGCATGACCAATCTTCCTTAACTCGCTTCATCCT-3'
24	5'-Amine-CAACAATATACTTCAGCATCCTGAGGCTTCATCGCG-3'
25	5'-Amine-TCTTCAGTACCGTACCGACCTTGAATTATAGCTTGT-3'
26	5'-Amine-ATGAGACAGTGCATTGTCGGTCGCACCGTGTTCTGA-3'
27	5'-Amine-GTCGATAAGTATATGGACGGTATTCGATATCTTGAT-3'
28	5'-Amine-TTAGGCGTAGATGCAGCTGGCACCTACAACGCCATG-3'
29	5'-Amine-GTCAGGCATAGAGATAGAACTCAGAGGCACTGGTAT-3'
30	5'-Amine-TTGCTGTCGAGTCACGTGATCAGACATTGTTGACCG-3'
31	5'-Amine-CAGCACGTCCGTTATCTGACCACATGATCTGATCTA-3'
32	5'-Amine-ATAGTACAGAATCGTGCCGCGCTATTCACGCGTTCG-3'

33	5'-Amine-TGAGATTATCAACGCGGTTCCATAGATGTACAGTTC-3'
34	5'-Amine-TGATAATCTTAGGAATTACCAGTAATGCTTAATCGT-3'
35	5'-Amine-ACGATATGAATTACACAGATCATTCTGCCATAACGG-3'
36	5'-Amine-CTTGCTTCCTAGAGTGTCGACGTGTGCTATCACCGC-3'
37	5'-Amine-TTCTTCCGCAATGCGGCCACGTGACTCGAGATTCGA-3'
38	5'-Amine-ATATTGGCGCGGCAGAATCCGGTAAGTGTAACAGCA-3'
39	5'-Amine-GTAATTGTTGAAGAGATCTCCAGAACACTCGCTATG-3'
40	5'-Amine-ATTACGGAGCGGCTCTCTTCTATGTTCACTACCTCT-3'

Table 2. List of sequences of 14 partner strands studied. The italic letters in each oligonucleotide indicate 5' overhanging single stranded bases.

Spot	Sequence
2	5'-*GCATGA*GTCTCCATCTGGTGTTAACC-3'
5	5'-*GTTGGA*CCAACAAGTGTCGCTAGAGG-3'
7	5'-*GAACGC*GGACCTGCATGTGCGTATTC-3'
8	5'-*ATAATT*CTACCTTGCTAAGTGTACAA-3'
10	5'-*CCTGGA*AGCAGGATGAATCTTGATAT-3'
12	5'-*GGCTGG*ATCTCCGCACAGCACCGCAC-3'
15	5'-*TGTTCG*AGTCTATCTCACGTGCGCGT-3'
18	5'-*GTCCAA*GACGAAGAGTTAACGCATAG-3'
21	5'-*ATAGTT*CTTCGGAACCTCTCACAGTC-3'
24	5'-*ATCTTA*CGCGATGAAGCCTCAGGATG-3'
26	5'-*TCTACG*TCAGAACACGGTGCGACCGA-3'
31	5'-*CGATCG*TAGATCAGATCATGTGGTCA-3'
34	5'-*GCAGTC*ACGATTAAGCATTACTGGTA-3'
36	5'-*CGTATA*GCGGTGATAGCACACGTCGA-3'
38	5'-*GCTAAC*TGCTGTTACACTTACCGGAT-3'
